# Post-Surgical Remodeling of Circulating Monocytes Identifies CD86 Expression on Non-Classical Monocytes as a Prognostic Indicator in Pancreatic Ductal Adenocarcinoma

**DOI:** 10.3390/ijms27115012

**Published:** 2026-06-01

**Authors:** Lisa-Sophie Arnold, Anne Jacobsen, Isabelle Kuchenreuther, Johanne Mazurie, Finn Niklas Clausen, Melanie Litau, Sebastian Klöckner, Franziska Czubayko, Bruno Leonardo Bancke Laverde, Yazan Amin, Nadine Weisel, Bettina Klösch, Susanne Merkel, Maximilian Brunner, Christian Krautz, Robert Grützmann, Anke Mittelstädt, Georg F. Weber, Paul David

**Affiliations:** 1 Department of Surgery, University Hospital Erlangen, 91054 Erlangen, Germany; lisa-sophie.arnold@fau.de (L.-S.A.); anne.jacobsen@uk-erlangen.de (A.J.); johanne.mazurie@fau.de (J.M.); finn.clausen@fau.de (F.N.C.); melanie.litau@uk-erlangen.de (M.L.); sebastian.kloeckner@fau.de (S.K.); franziska.czubayko@uk-erlangen.de (F.C.); bruno.banckelaverde@uk-erlangen.de (B.L.B.L.); yazan.alhajamin@uk-erlangen.de (Y.A.); nadine.weisel@uk-erlangen.de (N.W.); bettina.kloesch@uk-erlangen.de (B.K.); susanne.merkel@uk-erlangen.de (S.M.); maximilian.brunner@uk-erlangen.de (M.B.); christian.krautz@uk-erlangen.de (C.K.); robert.gruetzmann@uk-erlangen.de (R.G.); anke.mittelstaedt@uk-erlangen.de (A.M.); 2 Faculty of Medicine, Friedrich-Alexander-Universität Erlangen-Nürnberg (FAU), 91054 Erlangen, Germany

**Keywords:** CD86, non classical monocytes, monocytes, PDAC, postoperative, surgery

## Abstract

Circulating myeloid cells are critical regulators of pancreatic ductal adenocarcinoma (PDAC) progression. However, their postoperative dynamics and clinical relevance remain poorly defined. In a prospective longitudinal study, blood was collected from PDAC patients prior to surgery and on postoperative day 7. Flow cytometry was used to characterize monocytes, including classical (CM), intermediate (IMM), and non-classical (NCM) subsets, along with regulatory and co-stimulatory molecules (CD71, CD40, CD141, CD80, CD86, PD-L1). Cytokine levels (IL-6, IL-10, TNF-α, IL-1β) were quantified by ELISA and correlated with clinical parameters and survival. Total monocytes and CM increased significantly at day 7, whereas IMM and NCM decreased. CD71, CD141, CD80, and CD86 expression were significantly altered across subsets, with the most pronounced reduction observed in CD86 expressing NCM. CD86 expressing NCM correlated inversely with systemic bilirubin but not CEA, lymphocytes, thrombocytes, or hospital stay. IL-6 and IL-10 increased postoperatively; IL-10 showed a tendency toward inverse correlation with CD86^+^ NCM. Low CD86 expression on NCM at day 7 was associated with reduced survival and higher relapse probability. CD86 expression on NCMs is profoundly reduced after PDAC surgery and serves as a prognostic biomarker linked to inflammation, bilirubin metabolism, survival, and recurrence. Postoperative monocyte profiling may improve risk stratification and inform early clinical decision-making.

## 1. Introduction

Pancreatic ductal adenocarcinoma (PDAC) remains one of the most lethal malignancies, with a 5-year survival rate below 12% and an incidence that continues to rise worldwide [1,2,3]. Most patients present with advanced disease, and even among those eligible for surgical resection—the only potentially curative treatment—recurrence rates remain exceedingly high [4,5]. These clinical challenges underscore the need for improved understanding of systemic and perioperative immunobiology, which may shape both postoperative recovery and long-term oncologic outcomes.

An increasing body of evidence demonstrates that circulating myeloid cells, including monocytes and neutrophils, play essential roles in PDAC progression. These cells regulate inflammation, tumor–immune interactions, stromal remodeling, and systemic metabolic adaptations [6,7,8,9,10,11]. Our previous work has shown that circulating monocytes in PDAC exhibit altered phenotypes and functional profiles while CD71-expressing neutrophils contribute to disease aggressiveness [6,7]. More recently, HVEM-expressing monocytes were identified as correlates of advanced disease features [12]. Together, these findings highlight that systemic myeloid reprogramming is a hallmark of PDAC, reflecting underlying tumor biology and potentially influencing patient prognosis.

Despite these insights, the postoperative immune response remains insufficiently characterized. Major abdominal surgery induces a complex sequence of immunological events, beginning with a strong inflammatory response to tissue injury and followed by a compensatory anti-inflammatory phase [13,14,15,16,17]. This biphasic reaction may transiently impair cellular immunity, including the activity of monocytes—cells that play key roles in antigen presentation, cytokine production, and immune regulation [18,19]. Because PDAC patients are at high risk of early recurrence, understanding how monocyte subsets respond after tumor removal may provide critical insights into postoperative cancer biology [4].

Monocytes comprise three major subsets—classical monocytes (CMs), intermediate monocytes (IMMs), and non-classical monocytes (NCMs)—each characterized by distinct roles in immunity, vascular surveillance, and tissue repair [20,21]. NCM, in particular, contribute to endothelial patrolling and coordination of adaptive immune responses [22,23]. Surface molecules such as CD80 and CD86 (co-stimulatory ligands), CD141 (regulatory receptor), and PD-L1 (immune inhibitory ligand) govern monocyte–T cell interactions and influence downstream immune activation [24,25]. Alterations in the expression of these molecules may indicate shifts in postoperative immune competence or the emergence of immunosuppressive signatures [15,24,26,27,28,29,30,31].

The postoperative period represents both a window of vulnerability and a window of opportunity [32]. Immune dysregulation following surgery may facilitate micrometastatic growth, while this same timeframe offers clinically practical access to immune monitoring. Postoperative day 7 is particularly meaningful, as most PDAC patients stabilize sufficiently for hospital discharge, making it a standardized and feasible point for immunological assessment [33].

In this longitudinal study, we analyzed circulating monocytes before surgery and on postoperative day 7 in patients with PDAC. We quantified monocyte subsets, examined phenotypic markers associated with activation and immunoregulation, and evaluated relationships with clinical parameters and postoperative cytokine levels. Finally, we assessed whether postoperative monocyte signatures predict survival and relapse.

## 2. Results

### 2.1. Post-Surgical Expansion of Total and Classical Monocytes, with Reduction of NCM and IMM

**Figure 1:**
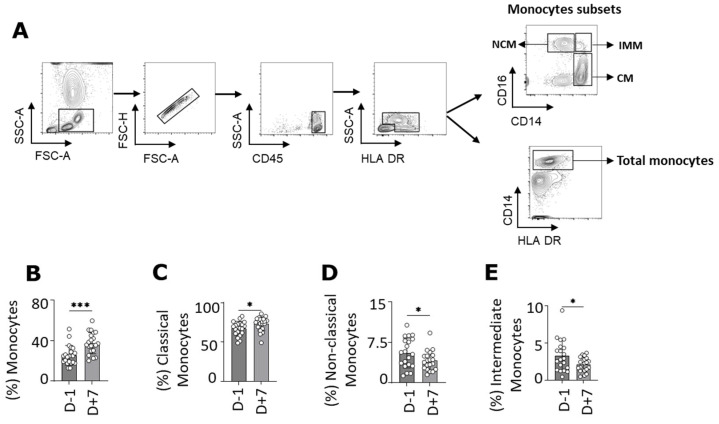
Flow cytometric gating strategy and postoperative changes in circulating monocyte subsets. (**A**) Sequential gating strategy used to identify monocytes, classical monocytes (CMs; CD14^++^CD16^−^), intermediate monocytes (IMMs; CD14^++^CD16^+^), and non-classical monocytes (NCMs; CD14^+^CD16^++^). (**B**–**E**) Frequencies of total monocytes, CM, IMM, and NCM in peripheral blood before surgery and on postoperative day 7. * *p* < 0.05; *** *p* < 0.001.

To characterize circulating monocyte subsets before and after surgery, we performed multistep flow cytometry as previously [6,12]. Cells were first gated according to FSC-A/SSC-A characteristics to include mononuclear leukocytes while excluding debris and granulocytes. Singlets were subsequently identified using FSC-H/FSC-A, followed by selection of CD45^+^ leukocytes and HLA-DR^+^ cells. From the HLA-DR^+^ population, two downstream analyses were performed: total monocytes were quantified using CD14/HLA-DR expression, whereas monocyte subsets were defined in parallel using CD14/CD16 expression. Classical monocytes were defined as CD14^++^CD16^−^, intermediate monocytes as CD14^++^CD16^+^, and non-classical monocytes as CD14^+^CD16^++^. Flow cytometric profiling revealed distinct postoperative alterations in circulating monocyte populations (Figure 1A). Total monocyte frequencies were significantly elevated on postoperative day 7 compared with pre-surgery levels, indicating enhanced myeloid mobilization following surgical intervention (Figure 1B). This increase was primarily driven by a significant increase in the frequencies of classical monocytes (CMs), which are typically associated with acute inflammatory responses and early tissue repair processes (Figure 1C).

In contrast to the expansion of CMs, both the frequencies of intermediate monocytes (IMMs) and non-classical monocytes (NCMs) were significantly reduced at postoperative day 7 (Figure 1D,E). As IMM and NCM represent more differentiated and functionally specialized subsets involved in antigen presentation, vascular patrolling, and immune regulation, their postoperative decline suggests a temporary suppression or redistribution of these populations during early recovery.

Collectively, these findings demonstrate a postoperative shift toward a classical-monocyte-dominant inflammatory profile, accompanied by a loss of more differentiated monocyte subsets. This pattern reflects the acute immunological response to surgical trauma and may have implications for postoperative immune competence and patient outcomes.

### 2.2. Altered Expression of Co-Stimulatory and Regulatory Molecules on Monocyte Subsets

**Figure 2:**
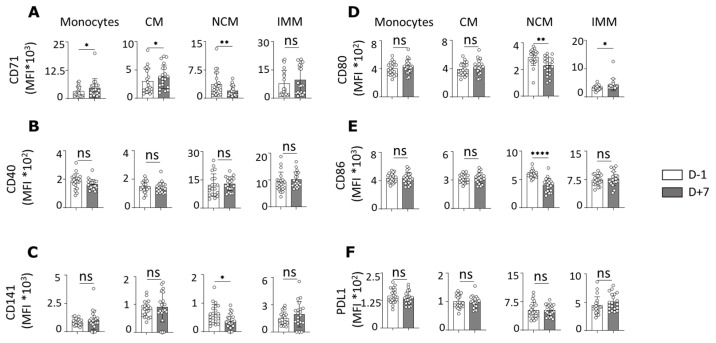
Postoperative modulation of activation and immunoregulatory markers on monocyte subsets in PDAC patients. (**A**) MFI of CD71 on total monocytes, CM, NCM and IMM before surgery (D-1) and on postoperative day 7 (D+7). (**B**) MFI of CD40 on total monocytes, CM, NCM and IMM before surgery (D-1) and on postoperative day 7 (D+7). (**C**) MFI of CD141 on total monocytes, CM, NCM and IMM before surgery (D-1) and on postoperative day 7 (D+7). (**D**) MFI of CD80 on total monocytes, CM, NCM and IMM before surgery (D-1) and on postoperative day 7 (D+7). (**E**) MFI of CD86 on total monocytes, CM, NCM and IMM before surgery (D-1) and on postoperative day 7 (D+7). (**F**) MFI of PDL1 on total monocytes, CM, NCM and IMM before surgery (D-1) and on postoperative day 7 (D+7). ns—non significant; * *p* < 0.05; ** *p* < 0.01 and **** *p* < 0.0001.

To further characterize the phenotypic changes occurring after surgery, we assessed the mean fluorescence intensity (MFI) of key molecules involved in monocyte activation and immunoregulation, including CD71, CD40, CD141, CD80, CD86, and PD-L1 across all monocyte subsets (total monocytes, CM, IMM, and NCM). Analysis revealed that CD71, CD141, CD80, and CD86 exhibited significant postoperative alterations, with expression patterns differing between monocyte subsets when comparing pre-surgery values to postoperative day 7 (Figure 2A,C–E).

In contrast, CD40 and PD-L1 did not display consistent postoperative changes, suggesting that inhibitory signaling pathways mediated by these molecules remain relatively stable during early recovery (Figure 2B,F).

Among all markers analyzed, the most striking change was observed in CD86 expression on non-classical monocytes (NCMs). CD86 MFIs on NCMs were significantly reduced on postoperative day 7, coinciding with the substantial numerical decline of this subset. Given the critical role of CD86 as a co-stimulatory molecule for T-cell activation, this marked postoperative reduction indicates that surgical inflammation may selectively dampen the immunostimulatory capacity of NCMs.

Together, these findings demonstrate that early postoperative immune remodeling involves not only shifts in monocyte subset composition but also targeted suppression of co-stimulatory phenotypes, particularly within the NCM compartment.

### 2.3. CD86 Expression on NCM Is Selectively Associated with Systemic Bilirubin Levels

To investigate whether postoperative CD86 expression on non-classical monocytes (NCMs) was associated with clinically relevant biochemical and hematological alterations, correlation analyses were performed using postoperative day 7 values across routinely assessed clinical parameters.

**Figure 3:**
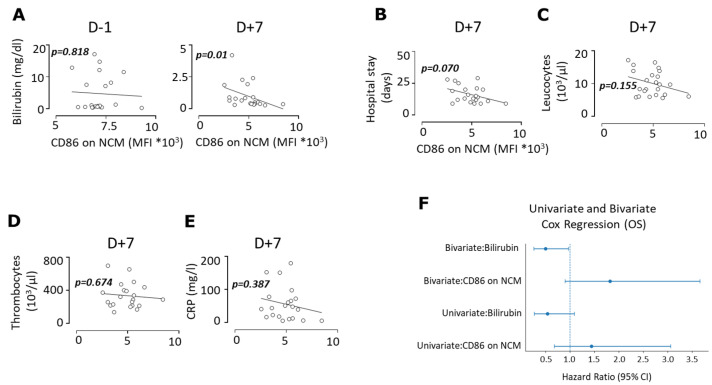
Correlation of postoperative CD86 MFI on NCMs with clinical and laboratory parameters in PDAC patients. (**A**) Scatter plot showing correlation between preoperative (D-1) and postoperative day 7 CD86 expression on NCMs and systemic bilirubin levels. (**B**). Correlation analyses between CD86 expression on NCMs and PDAC patient’s hospital stay (days). (**C**–**E**) Correlation analyses between CD86 expression on NCMs and postoperative lymphocyte counts, thrombocyte counts, and CRP levels. (**F**) Forest Plot showing the Univariate and Bivariate Cox regression plot of CD86 expression on NCMs and bilirubin on day 7.

A significant inverse correlation was observed between postoperative CD86 expression on NCM, measured as CD86 MFI and systemic bilirubin levels, whereas no association was detected preoperatively (day-1) (Figure 3A). Patients with elevated postoperative bilirubin—reflecting impaired hepatic recovery, cholestatic stress, or postoperative metabolic burden—consistently exhibited reduced CD86 expression on NCM. These findings suggest a potential association between postoperative monocyte remodeling and hepatobiliary physiology. Given the role of NCM in vascular surveillance and tissue homeostasis, reduced CD86 expression on NCM may reflect impaired monocyte maturation or altered immune–liver communication during postoperative inflammatory stress. However, this association should be interpreted cautiously due to the exploratory nature of the analysis and the limited cohort size.

An additional inverse trend was observed between postoperative CD86 expression on NCMs and duration of hospital stay (*p* = 0.07) (Figure 3B), indicating that patients with lower day-7 CD86 expression on NCMs tended to require prolonged postoperative recovery. In contrast, no significant correlations were identified between CD86 expression on NCMs and lymphocyte counts, thrombocyte counts, or CRP levels (Figure 3C–E), suggesting that the observed changes do not simply reflect generalized postoperative inflammation or hematologic recovery.

To evaluate the prognostic significance of these findings, Cox proportional hazards analyses were performed. In univariate analysis, postoperative CD86 expression on NCMs were significantly associated with overall survival (HR 0.9993, 95% CI 0.9986–0.9999, *p* = 0.041), whereas bilirubin showed only a non-significant trend (HR 1.82, 95% CI 0.90–3.66, *p* = 0.094). In the bivariate model including both CD86 expression on NCMs and bilirubin, the prognostic effect of CD86^+^ NCM was attenuated but remained evident as a strong trend (HR 0.9994, 95% CI 0.9987–1.0001, *p* = 0.084), while bilirubin lost prognostic significance (HR 1.44, 95% CI 0.68–3.06, *p* = 0.34) (Figure 3F; Table S1). These findings suggest partial overlap between postoperative hepatobiliary stress and monocyte immune remodeling while supporting continued prognostic relevance of CD86 expression on NCMs beyond bilirubin alone.

To further address potential perioperative confounding factors, exploratory stratified analyses were performed according to biliary stent status, neoadjuvant therapy, postoperative pancreatic fistula (POPF), and hepatic steatosis. Across evaluable strata, the inverse bilirubin–CD86 expression on the NCM relationship remained directionally consistent (Figure S1). Kaplan–Meier analyses further demonstrated that low postoperative CD86 expression on NCM frequencies was associated with inferior overall survival (OS) and relapse-free survival (RFS) across several clinically relevant subgroups, particularly in non-stented patients, patients without neoadjuvant therapy, and patients without clinically relevant POPF (Figures S2–S5). Subgroup Cox analyses similarly demonstrated persistent protective associations of higher CD86 expression on NCMs with reduced risks of death and relapse (Figure S6; Table S2). Owing to limited subgroup sizes and event numbers, these analyses should be considered exploratory.

Collectively, these findings identify CD86 expression on NCMs as a distinct postoperative immunological biomarker associated with hepatobiliary stress, postoperative recovery, survival, and relapse. The retained prognostic signal in bivariate and stratified analyses supports the concept that CD86 expression on NCMs reflects an integrated state of postoperative immune competence and systemic physiological adaptation in PDAC patients.

### 2.4. Post-Surgical IL-6 and IL-10 Increase and IL-10 Tends to Inversely Correlate with CD86 Expression on NCMs

**Figure 4:**
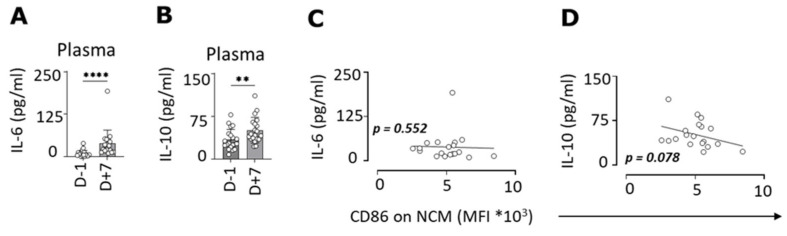
Postoperative cytokine changes and their association with CD86^+^ non-classical monocytes in PDAC patients. (**A**,**B**) Plasma levels of IL-6 and IL-10 measured before surgery (Pre) and on postoperative day 7 (D7). (**C**) Correlation between postoperative IL-6 levels and CD86 expression on NCMs at day 7. (**D**) Correlation between postoperative IL-10 levels and CD86 expression on NCMs at day 7. ** *p* < 0.01 and **** *p* < 0.0001.

To examine whether postoperative cytokine responses were associated with changes in monocyte activation, we quantified circulating IL-6, IL-10, TNF-α, and IL-1β levels before surgery and on postoperative day 7. IL-6 levels were significantly elevated on day 7 compared with pre-surgery, reflecting the acute inflammatory response to major abdominal surgery (Figure 4A). IL-10, a key anti-inflammatory cytokine, was likewise significantly increased postoperatively, indicating activation of compensatory anti-inflammatory pathways during early recovery (Figure 4B).

In contrast, TNF-α and IL-1β showed no significant differences between pre-surgery and day 7, suggesting that these classical pro-inflammatory mediators do not contribute substantially to the early postoperative immune profile in this exploratory patient cohort (Figure S7). Based on the absence of postoperative modulation, we did not perform correlation analyses between CD86 expression on NCMs and either TNF-α or IL-1β.

Correlation analyses revealed that postoperative IL-6 levels did not significantly associate with the CD86 expression on NCMs (Figure 4C). However, IL-10 demonstrated a trend toward an inverse relationship with CD86 expression on NCMs, suggesting that stronger postoperative anti-inflammatory signaling may be accompanied by reduced co-stimulatory capacity within the NCM compartment (Figure 4D). These findings indicate that the postoperative cytokine milieu, particularly IL-10, may contribute to the modulation of CD86 expression on NCM or reflect a broader immunoregulatory shift occurring during the early postoperative period.

### 2.5. CD86 Expression on NCMs at Day 7 Was Associated with Survival and Relapse in PDAC Patients

**Figure 5:**
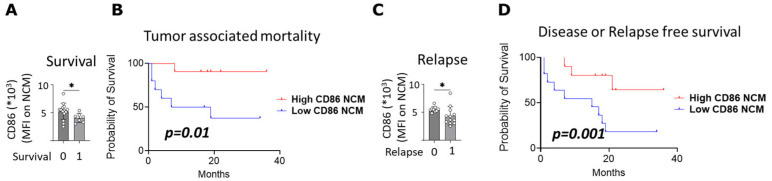
Postoperative CD86 expression on NCMs is associated with survival and relapse in PDAC patients. (**A**) Comparison of CD86 expression on NCMs at postoperative day 7 between PDAC patients who survived versus those who later died. (**B**) Kaplan–Meier overall survival curves stratified by high versus low postoperative CD86 expression on NCMs. (**C**) CD86 expression on NCM frequencies at day 7 in relapse-free patients compared with patients who relapsed or died. (**D**) Kaplan–Meier relapse-free survival curves demonstrating higher relapse probability among patients with low postoperative CD86 expression on NCMs. * *p* < 0.05.

To determine whether postoperative CD86 expression on NCMs is associated with patient outcome, we analyzed their frequencies on day 7 after surgery in relation to overall survival and relapse status. When patients were stratified into survivors and non-survivors, we observed that individuals who subsequently died exhibited significantly lower CD86 expression on NCMs at postoperative day 7 compared with those who remained alive (Figure 5A). This indicates that reduced postoperative CD86 expression on NCM is associated with poor overall survival.

Consistent with this observation, Kaplan–Meier survival analysis demonstrated that patients with low CD86 expression on NCMs had markedly shorter survival times compared with those with high CD86 expression on NCMs (Figure 5B). These findings identify postoperative CD86 expression on NCMs as a prognostic marker for long-term outcome.

Next, we evaluated whether postoperative CD86 expression on NCM was associated with tumor relapse. Patients were grouped into relapse/death versus no relapse categories. Similar to survival, patients who relapsed or died displayed significantly lower CD86 expression on NCMs on postoperative day 7 relative to relapse-free patients (Figure 5C). Survival analysis confirmed that low CD86 expression on NCM was associated with increased relapse risk and shortened relapse-free survival (Figure 5D). Together, these data demonstrate that reduced postoperative CD86 expression on NCM is linked to both poor survival and higher likelihood of disease recurrence. Overall, these data indicate that postoperative CD86 expression on NCMs represents a clinically relevant immune biomarker, with reduced levels associated with both poor survival and higher relapse probability in PDAC patients.

## 3. Discussion

Pancreatic ductal adenocarcinoma (PDAC) remains one of the most aggressive malignancies, largely due to its late diagnosis, profound immunosuppressive microenvironment, and high relapse rate even after successful tumor resection [1,2,4,5,34,35]. Understanding perioperative immunodynamics is thus critical for improving prognostic assessment and guiding early post-surgical interventions [36,37]. This study provides new evidence that circulating monocytes undergo substantial remodeling by postoperative day 7 and identifies CD86 expression on NCMs as a biomarker linked to inflammation, clinical chemistry, and patient outcome [11,18,38].

Our findings demonstrate that post-surgical immune changes extend beyond classical inflammatory responses. The observed expansion of classical monocytes (CMs) at day 7 is consistent with emergency myelopoiesis triggered by surgical stress, tissue damage, and acute-phase cytokines [39,40,41,42]. Classical monocytes are rapidly mobilized to support inflammation, wound healing, and pathogen defense. In contrast, the significant decline in non-classical and intermediate monocytes (NCMs, IMMs) is notable because these subsets play essential roles in vascular patrolling, tissue repair, and immunoregulation [22,43,44]. Their depletion may reduce the capacity to resolve inflammation or engage in anti-tumor surveillance in the immediate postoperative period.

Among all markers analyzed, CD86 MFI on NCM showed the most substantial reduction. CD86 is a costimulatory ligand required for T-cell activation through CD28 signaling [45,46]. Diminished CD86 expression may therefore impair the ability of NCM to support adaptive immunity during a period when immune reconstitution is critical [26,47]. This suppression could result from systemic anti-inflammatory pathways activated after major surgery, including IL-10, which we found elevated at day 7 and inversely trending with CD86 expression on NCM [48,49].

One of the most clinically relevant findings of this study was the inverse association between CD86 expression on NCMs and postoperative bilirubin levels [50,51,52]. Although bilirubin elevation is common in PDAC because of preoperative biliary obstruction, postoperative bilirubin may additionally reflect delayed hepatobiliary recovery, metabolic stress, cholestasis, or subclinical postoperative complications. Patients with elevated bilirubin consistently exhibited reduced CD86 expression on NCMs, suggesting a close relationship between postoperative monocyte remodeling and hepatobiliary physiology [53,54,55]. Given the central role of the liver in regulating innate immune homeostasis, these findings raise the possibility that impaired monocyte–liver crosstalk or prolonged postoperative metabolic stress contributes to suppression of co-stimulatory NCM phenotypes [32,56]. The additional inverse trend observed between CD86 expression on NCMs and duration of postoperative hospital stay further supports this interpretation, as patients with lower CD86 expression on NCMs tended to require prolonged postoperative recovery.

Because bilirubin itself may influence postoperative outcome, an important question was whether CD86 expression on NCMs simply reflected hepatobiliary dysfunction rather than representing an independent immunological biomarker. To address this, we performed both univariate and bivariate Cox proportional hazards analyses. In univariate analysis, postoperative CD86 expression on NCMs was significantly associated with overall survival, whereas bilirubin demonstrated only a non-significant trend. In the bivariate model including both CD86 expression on NCM and bilirubin, the prognostic effect of CD86 expression on NCM was attenuated but remained evident as a strong trend, while bilirubin lost prognostic significance entirely [51,57,58]. These findings suggest partial biological overlap between postoperative hepatobiliary stress and monocyte immune remodeling, while simultaneously arguing against bilirubin alone accounting for the observed prognostic associations.

To further evaluate potential confounding pathways, exploratory stratified analyses were performed according to biliary stent status, neoadjuvant therapy, postoperative pancreatic fistula (POPF), and hepatic steatosis [55,59,60,61,62]. Across evaluable strata, the inverse relationship between bilirubin and CD86 expression on NCMs remained directionally consistent. Moreover, low postoperative CD86 expression on NCMs remained associated with inferior overall survival and relapse-free survival across multiple clinically relevant subgroups, including non-stented patients, patients without neoadjuvant therapy, and patients without clinically relevant POPF. Subgroup Cox analyses similarly demonstrated persistent protective associations of higher CD86 expression on NCMs with both overall and relapse-free survival in major evaluable strata. Although these analyses remain exploratory because of the limited cohort size, they argue against the CD86 expression on NCMs signal being driven solely by a single confounding clinical factor.

Importantly, the prognostic associations identified here highlight a clinically meaningful observation: patients with low CD86 expression on NCM at day 7 showed significantly worse survival and higher risk of relapse [11,32,53,54]. This suggests that early postoperative immune recovery—specifically the reconstitution of co-stimulatory non-classical monocytes may set the trajectory for long-term cancer control. These findings complement our earlier reports on myeloid involvement in PDAC progression and highlight the postoperative window as both a vulnerable and actionable period.

From a translational perspective, this study identifies CD86 expression on NCMs as an accessible, blood-based biomarker measurable with routine flow cytometry. Its association with survival and relapse suggests potential utility in patient stratification, personalized follow-up intensity, or early immunotherapeutic intervention [50,51,52]. Even if CD86 expression on NCMs partially reflects postoperative hepatobiliary stress, this may strengthen rather than diminish their translational value by positioning them as an integrated biomarker of postoperative immune competence and systemic physiological adaptation.

Several limitations of this study should be acknowledged. The cohort size was relatively small, limiting statistical power for extensive multivariable and subgroup analyses. In addition, a small subset of patients with high-grade IPMN was included alongside PDAC patients, introducing potential biological heterogeneity. Furthermore, although the stratified analyses argue against a single confounding mechanism, larger independent cohorts will be necessary to validate the prognostic independence of CD86 expression on NCMs and to better define the mechanistic relationship between postoperative hepatobiliary stress and monocyte dysfunction. Therefore, the observed association between CD86 expression on NCM and survival or relapse should be interpreted as exploratory and hypothesis-generating rather than as evidence of an independent prognostic effect. Because of the limited cohort size, demographic subgroup analyses, including sex-specific and age-associated immune differences, could not be performed robustly. Future larger-scale studies are needed to evaluate the generalizability of CD86 expressing NCM as a biomarker across diverse patient populations.

In summary, our study demonstrates that the early postoperative period in PDAC is characterized by profound immune remodeling, with suppression of CD86 expression on non-classical monocytes emerging as a central feature associated with inflammatory cytokines, bilirubin metabolism, survival, and relapse. Monitoring CD86 expression on NCMs may therefore provide clinically relevant insight into postoperative immune competence and represents a promising approach for refining prognostic assessment and understanding the immunological determinants of postoperative outcome in PDAC.

## 4. Materials and Methods

### 4.1. Patient Cohort and Sample Collection

Preoperative (D-1) and postoperative (D+7) peripheral blood samples were collected from patients aged ≥40 years of both sexes who underwent elective surgery for suspected PDAC at the Department of Surgery, University Hospital Erlangen, Germany. Postoperative histopathological evaluation identified 18 patients with pancreatic ductal adenocarcinoma (PDAC) and 3 patients with high-grade intraductal papillary mucinous neoplasm (IPMN). Because high-grade IPMN represents an advanced premalignant pancreatic lesion with substantial inflammatory and surgical overlap with PDAC, these patients were included in the longitudinal perioperative immune analyses (Table S3). The study was conducted in accordance with the Declaration of Helsinki and approved by the Institutional Review Board of the University Hospital Erlangen (approval number 180_19 B, 14 June 2019). Written informed consent was obtained from all participants or their legal representatives prior to enrollment.

### 4.2. Sample Preparation

Peripheral blood was collected in 7.5 mL EDTA tubes (Cat. No. 01.1605.001, Sarstedt, Nümbrecht, Germany) and was processed within 2 h of collection. Samples were first centrifuged at 350× *g* for 10 min at room temperature. Plasma was carefully aspirated and stored at −80 °C for subsequent cytokine analysis. The remaining cellular fraction was subjected to red blood cell lysis using a 1:10 dilution of 1× RBC Lysis Buffer (Cat. No. 555899, BD Biosciences, Franklin Lakes, NJ, USA) and incubated for 15 min at room temperature. Following lysis, samples were centrifuged at 350× *g* for 5 min, and the cell pellet was resuspended in 50 mL of 1× PBS (Cat. No. 14190169, Gibco, Waltham, MA, USA). After an additional centrifugation step (350× *g*, 5 min), cells were resuspended in FACS buffer (1X PBS with 1% FBS (Cat. No. A3160802, Gibco), 0.5% BSA (Cat. No. A2153, Sigma Aldrich, St. Louis, MO, USA), and 2 mM EDTA (Cat. No. AM9260G, Invitrogen, Waltham, MA, USA). The cell suspension was transferred into 96-well plates (Cat. No. 651101, Greiner Bio-One, Kremsmünster, Austria), washed with FACS buffer, and incubated with fluorochrome-conjugated antibodies for 20 min at 4 °C. After staining, cells were washed twice with FACS buffer and finally resuspended for acquisition. Flow cytometric analysis was performed on a BD Celesta™ analyzer (BD Biosciences) using BD FACSDiva™ Software v8.0.1.1, and data were analyzed with FlowJo™ Software v10.9.0 (FlowJo LLC, Ashland, OR, USA).

### 4.3. Flow Cytometry and Monocyte Gating

Cells were stained with fluorescence-conjugated antibodies targeting major monocyte markers and regulatory/stimulatory molecules: anti-CD16-FITC (Cat-No. 555406, BD Biosciences, Franklin Lakes, NJ, USA); anti-CD40-PE (Cat-No. 313006, Biolegend, San Diego, CA, USA); anti-PDL1-PE-Dazzle (Cat-No. 563742); anti-HLA DR-PeCy5 (Cat-No. 555813, BD Biosciences, Franklin Lakes, NJ, USA); anti-CD80-BUV395 (Cat-No. 565210, BD Biosciences, Franklin Lakes, NJ, USA); anti-CD14-BUV737 (Cat-No. 612763, BD Biosciences, Franklin Lakes, NJ, USA); anti-CD45-BV421 (Cat-No. 304022, Biolegend, San Diego, CA, USA); anti-CD86-BV510 (Cat-No. 563461, BD Biosciences, Franklin Lakes, NJ, USA); anti-CD71-BV650 (Cat-No.743307, BD Biosciences, Franklin Lakes, NJ, USA); anti-CD141-BV786 (Cat-No. 344116, Biolegend, San Diego, CA, USA). Total monocytes were identified by FSC/SSC morphology, HLA-DR positivity and CD14^+^. Subsets were defined as: classical monocytes (CMs): CD14^++^CD16^−^; intermediate monocytes (IMMs): CD14^++^CD16; non-classical monocytes (NCMs): CD14^+^CD16^++^ were gated on HLA DR+ cells. Expression levels of regulatory and co-stimulatory markers were reported as median fluorescence intensity (MFI) or percentage positive cells.

### 4.4. Cytokine Quantification by Enzyme-Linked Immunosorbent Assays (ELISA)

ELISAs of plasma cytokines (IL-6 (Cat. No. 430504; Biolegend), IL-10 (Cat. No. 430604; Biolegend), TNF-α (Cat. No. 430204; Biolegend), IL-1β (Cat. No. 437004; Biolegend)) were performed according to the manufacturers’ instructions. Briefly, 100 µL of capture antibody diluted in coating buffer was added to each well of a 96-well plate and incubated overnight at 4 °C. Plates were then washed and blocked prior to the addition of 100 µL of appropriately diluted standards and plasma samples. Following a 2 h incubation at room temperature, plates were washed thoroughly. Next, 100 µL of biotinylated detection antibody diluted in assay diluent was added to each well and incubated for 1 h at room temperature. After additional washing steps, 100 µL of avidin–horseradish peroxidase (HRP) conjugate diluted in assay diluent was applied to each well and incubated for 30 min at room temperature. Following a final set of washes, 100 µL of TMB substrate solution was added and allowed to develop in the dark for 15 min. The reaction was stopped by adding 100 µL of stop solution, and absorbance was measured at 450 nm using a SpectraMax M3 ELISA plate reader (Molecular Devices, San Jose, CA, USA).

### 4.5. Clinical Data

Clinical parameters (lymphocyte counts, thrombocytes, bilirubin, hospitalization duration, relapse, survival) were collected from the internal medical records. Patients were followed until relapse or death.

### 4.6. Statistical Analysis

Data were assessed for distribution and analyzed using paired or unpaired tests as appropriate (Wilcoxon, Mann–Whitney). Correlations were assessed using Spearman rank testing. Kaplan–Meier survival analyses were performed using median CD86^+^ NCM values as cutoffs. *p* < 0.05 was considered statistically significant.

To evaluate the prognostic relevance of postoperative immune parameters, univariate and bivariate Cox proportional hazards regression analyses were performed. Variables included postoperative day 7 CD86 median fluorescence intensity (MFI) on non-classical monocytes (NCM), systemic bilirubin levels, hepatic steatosis, postoperative pancreatic fistula (POPF), neoadjuvant therapy, and biliary stent status. Hazard ratios (HR) with corresponding 95% confidence intervals (CI) were calculated. To improve interpretability, CD86 MFI on NCM was rescaled and analyzed per 1000-unit increase.

In univariate Cox analyses, each variable was evaluated independently for association with overall survival (OS) and relapse-free survival (RFS). Subsequently, exploratory bivariate Cox regression models were constructed to assess whether the prognostic association of CD86 MFI on NCM remained evident after adjustment for postoperative bilirubin and additional clinically relevant perioperative factors where statistically feasible. Due to the limited cohort size and event number, multivariable analyses were considered exploratory and hypothesis-generating.

Exploratory stratified analyses were additionally performed according to biliary stent status, neoadjuvant therapy, POPF status, and hepatic steatosis to assess the consistency of the observed associations across clinically relevant subgroups. A two-sided *p*-value < 0.05 was considered statistically significant.

## 5. Conclusions

This study reveals that PDAC surgery induces marked shifts in circulating monocytes, most notably a decline in CD86^+^ non-classical monocytes by postoperative day 7. Reduced levels of this subset were associated with higher bilirubin, increased IL-10, poorer survival, and greater relapse risk. These findings indicate that postoperative CD86^+^ NCM may serve as a practical blood-based biomarker to identify patients at elevated risk for adverse outcomes. Incorporating postoperative immune monitoring into clinical follow-up protocols could improve early risk stratification and guide timely management decisions.

## Data Availability

The original contributions presented in this study are included in the article/Supplementary Materials. Further inquiries can be directed to the corresponding authors.

## Supplementary Materials

The following supporting information can be downloaded at: https://www.mdpi.com/article/10.3390/ijms27115012/s1.

## Abbreviations

The following abbreviations are used in this manuscript:

PDAC	Pancreatic ductal adenocarcinoma
CM	Classical monocytes
IMM	Intermediate monocytes
NCM	Non-classical monocytes
HVEM	Herpes virus entry mediator
CD	Cluster of differentiation
PD-L1	Programmed death ligand 1
D-1	Day minus 1 (preoperative)
D+7	Day plus 7 (postoperative day 7)
HR	Hazard ratio
OS	Overall survival
RFS	Relapse-free survival
POPF	Postoperative pancreatic fistula
IL	Interleukin
TNF	Tumor necrosis factor
