# Supplementary material for: Post-Surgical Remodeling of Circulating Monocytes Identifies CD86 Expression on Non-Classical Monocytes as a Prognostic Indicator in Pancreatic Ductal Adenocarcinoma
Source: Int J Mol Sci. 2026 Jun 1;27(11):5012. doi: 10.3390/ijms27115012 (PMC13256181; doi:10.3390/ijms27115012)
Supplement: Supplementary file 1 [file ijms-27-05012-s001.zip › ijms-4346091-supplementary.pdf]

Figure S1

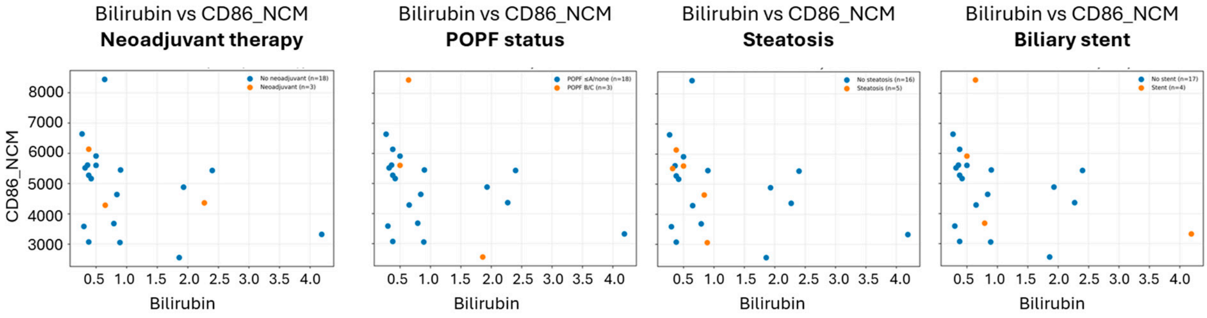

Figure S2

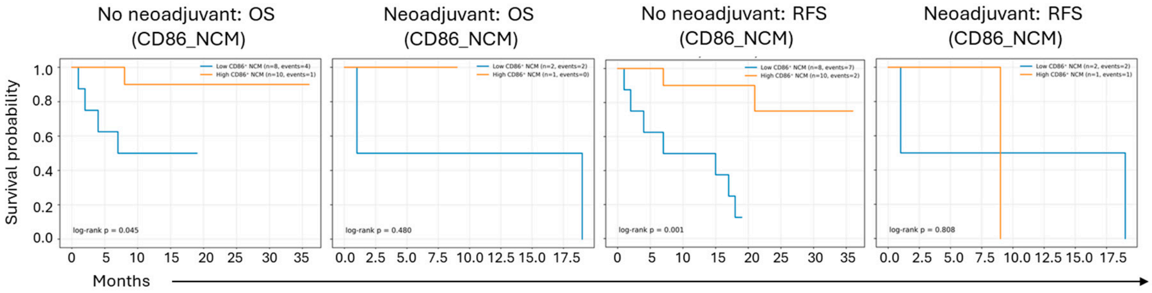

Figure S3

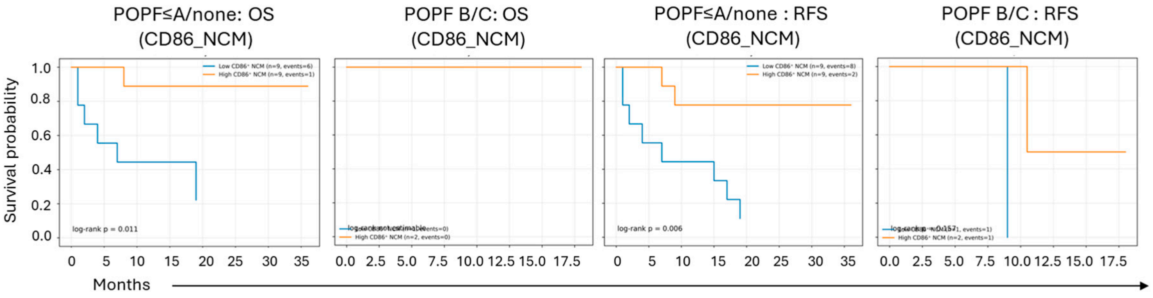

Figure S4

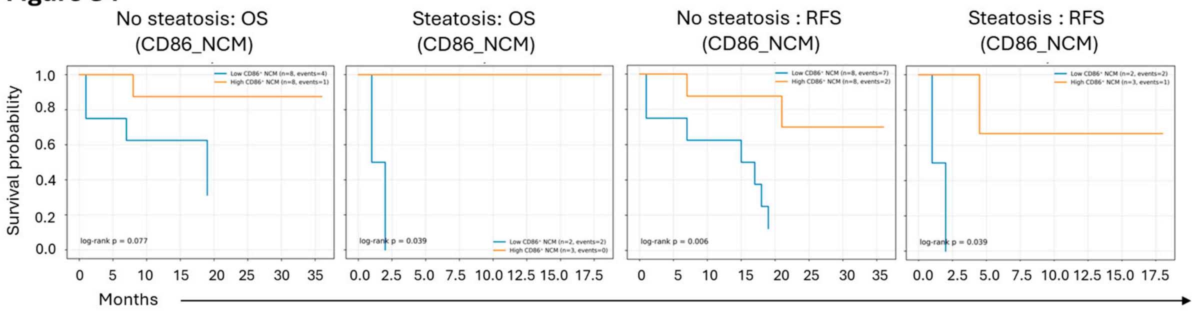

Figure S5

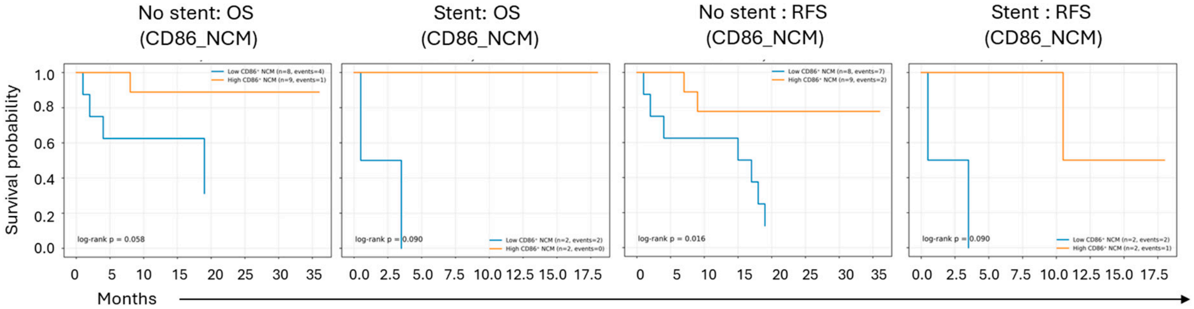

Figure S6

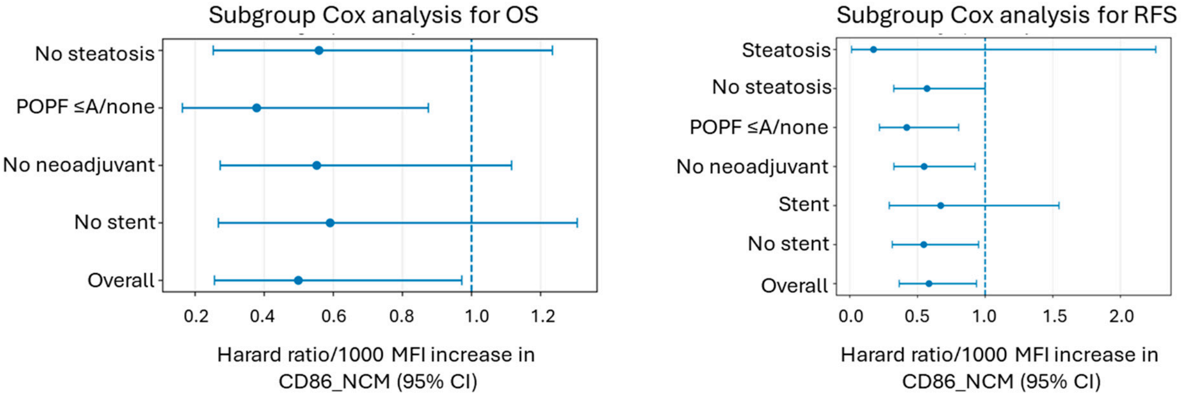

Figure S7

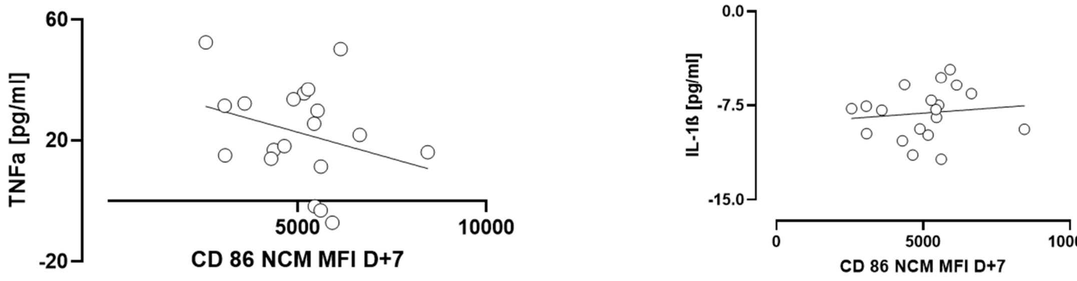

Table S1: Cox Regression

Variable,HR,CI Low,CI High,p-value
Univariate: CD86_NCM,0.4990165131631531,0.2562262789587606,0.9718654988138548,0.0409659091636852
Univariate: Bilirubin,1.8176123322959352,0.9024043294738016,3.6610136749240656,0.094422059576506
Bivariate: CD86_NCM,0.5448951909366315,0.27346138807040277,1.0857502450380638,0.0843333553624126
Bivariate: Bilirubin,1.4411316719362843,0.6778787996189173,3.0637637539710894,0.3422993165012606

Table S2a: Cleaned data

Disease	OS_time	OS_event	RFS_time	RFS_event	CD86_NCM	Bilirubin	Stent	Neoadjuvant	POPF	Steatosis	CD86_group	CD86_k
PDAC	36	0	36	0	5449	0.9	0	0	0	0	High $CD86^+$ NCM	5.449
IPMN	36	0	36	0	5911	0.5	1	0	0	0	High $CD86^+$ NCM	5.911
PDAC	36	0	36	0	5602	0.5	0	0	1	1	High $CD86^+$ NCM	5.602
PDAC	19	1	19	1	4363	2.271	0	1	0	0	Low $CD86^+$ NCM	4.363
PDAC	34	0	34	0	5165	0.42	0	0	0	0	High $CD86^+$ NCM	5.165

PDAC	1	1	1	1	3321	4.191	1	0	0	0	Low CD86$^+$ NCM	3.321
PDAC	4	1	4	1	4639	0.84	0	0	0	1	Low CD86$^+$ NCM	4.639
PDAC	22	0	22	0	5612	0.36	0	0	0	0	High CD86$^+$ NCM	5.612
PDAC	21	0	21	1	8442	0.64	1	0	1	0	High CD86$^+$ NCM	8.442
IPMN	17	0	17	1	3071	0.38	0	0	0	0	Low CD86$^+$ NCM	3.071
PDAC	1	1	1	1	4286	0.65	0	1	0	0	Low CD86$^+$ NCM	4.286
PDAC	2	1	2	1	3054	0.89	0	0	0	1	Low CD86$^+$ NCM	3.054
PDAC	7	1	7	1	3678	0.79	1	0	0	0	Low CD86$^+$ NCM	3.678
PDAC	18	0	18	1	2554	1.891	0	0	1	0	Low CD86$^+$ NCM	2.554
PDAC	19	0	19	0	4881	1.931	0	0	0	0	Low CD86$^+$ NCM	4.881
PDAC	19	0	19	0	5518	0.32	0	0	0	1	High CD86$^+$ NCM	5.518
PDAC	9	0	9	1	6135	0.38	0	1	0	1	High CD86$^+$ NCM	6.135
IPMN	18	0	18	0	6640	0.27	0	0	0	0	High CD86$^+$ NCM	6.641
PDAC	15	0	15	1	3585	0.3	0	0	0	0	Low CD86$^+$ NCM	3.585
PDAC	16	0	7	1	5432	2.412	0	0	0	0	High CD86$^+$ NCM	5.432
PDAC	8	1	16	0	5273	0.38	0	0	0	0	High CD86$^+$ NCM	5.273

Table S2b: Stratified correlations

Stratification	Stratum	n	Spearman rho	p-value
Biliary stent	No stent	17	-0.385750550112383	0.126203631616641
Biliary stent	Stent	4	-0.8	0.2
Neoadjuvant therapy	No neoadjuvant	18	-0.378099375309211	0.12184755392184
Neoadjuvant therapy	Neoadjuvant	3	-0.5	0.666666666666667
POPF status	POPF ≤A/none	18	-0.375388600205973	0.124764657705801
POPF status	POPF B/C	3	-0.5	0.666666666666667
Steatosis	No steatosis	16	-0.285504124387051	0.283768828623355
Steatosis	Steatosis	5	-0.7	0.188120404374187

Table S2c: Stratified KM

Endpoint	Stratification	Stratum	n	events	low_n	high_n	logrank_chi2	logrank_p
OS	Biliary stent	No stent	17	5	8	9	3.591	0.058
OS	Biliary stent	Stent	4	2	2	2	2.882	0.089
OS	Neoadjuvant therapy	No neoadjuvant	18	5	8	10	4.019	0.044

OS	Neoadjuvant therapy	Neoadjuvant	3	2	2	1	0.5	0.479
OS	POPF status	POPF ≤A/none	18	7	9	9	6.434	0.011
OS	POPF status	POPF B/C	3	0	1	2		
OS	Steatosis	No steatosis	16	5	8	8	3.136	0.076
OS	Steatosis	Steatosis	5	2	2	3	4.263	0.038
RFS	Biliary stent	No stent	17	9	8	9	5.851	0.015
RFS	Biliary stent	Stent	4	3	2	2	2.882	0.089
RFS	Neoadjuvant therapy	No neoadjuvant	18	9	8	10	10.937	0.000
RFS	Neoadjuvant therapy	Neoadjuvant	3	3	2	1	0.058	0.808
RFS	POPF status	POPF ≤A/none	18	10	9	9	7.553	0.005
RFS	POPF status	POPF B/C	3	2	1	2	2	0.157
RFS	Steatosis	No steatosis	16	9	8	8	7.517	0.006
RFS	Steatosis	Steatosis	5	3	2	3	4.263	0.038

Table S2d: Subgroup Cox

Endpoint	Subgroup	n	events	HR	CI_low	CI_high	p	estimable
OS	Overall	21	7	0.499	0.256	0.971	0.040	TRUE
OS	No stent	17	5	0.590	0.267	1.3	0.193	TRUE
OS	Stent	4	2					FALSE
OS	No neoadjuvant	18	5	0.551	0.272	1.116	0.098	TRUE

OS	Neoadjuvant	3	2					FALSE
OS	POPF ≤A/none	18	7	0.378	0.163	0.875	0.023	TRUE
OS	POPF B/C	3	0					FALSE
OS	No steatosis	16	5	0.558	0.252	1.234	0.149	TRUE
OS	Steatosis	5	2					FALSE
RFS	Overall	21	12	0.585	0.365	0.937	0.025	TRUE
RFS	No stent	17	9	0.546	0.314	0.952	0.032	TRUE
RFS	Stent	4	3	0.671	0.291	1.546	0.349	TRUE
RFS	No neoadjuvant	18	9	0.549	0.326	0.925	0.024	TRUE
RFS	Neoadjuvant	3	3					FALSE
RFS	POPF ≤A/none	18	10	0.420	0.219	0.805	0.008	TRUE
RFS	POPF B/C	3	2					FALSE
RFS	No steatosis	16	9	0.570	0.325	1.000	0.050	TRUE
RFS	Steatosis	5	3	0.175	0.0135	2.261	0.182	TRUE

Table S3: Characteristic feature of PDAC cohort

Patients		
Number		21
Mean age (in years[range])		70.4 [39-85]
Sex (male:female)		11:10
Tumor type	PDAC	18
	IPMN high grade	3
Pancreas tumor resected		21

Preoperative cholestasis	Yes	8
	No	13
Preoperative biliary stenting	Yes	4
	No	17
Resection type	PD	14
	DP	5
	TP	2
Pre-existing hepatic injury	Yes	10
	No	11
Neoadjuvant therapy	Yes	3
	No	18
Radiological steatosis from preoperative staging CT/MRI?	Yes	5
	No	16
Chronic liver disease	Yes	0
	No	21
Complications	0	4
	1	17
Clavien-dindo	0	3
	1	5
	2	6
	3a	5
	4a	2
POPF	0	18
	B	2
	BCL	1
